# Phase stability and electronic structure of iridium metal at the megabar range

**DOI:** 10.1038/s41598-019-45401-x

**Published:** 2019-06-20

**Authors:** V. Monteseguro, J. A. Sans, V. Cuartero, F. Cova, Igor A. Abrikosov, W. Olovsson, C. Popescu, S. Pascarelli, G. Garbarino, H. Johan M. Jönsson, T. Irifune, D. Errandonea

**Affiliations:** 1 Departamento de Física Aplicada-ICMUV, Universitat de València, MALTA Consolider Team, Edificio de Investigación, C/Dr. Moliner 50, 46100 Burjassot, Valencia, Spain; 2 European Radiation Synchrotron Facility, 38043 Grenoble, Cedex 9 France; 3 Instituto de Diseño para la Fabricación y Producción Automatizada, MALTA Consolider Team, Universitat Politècnica de València, 46022 Valencia, Spain; 4 Centro Universitario de la Defensa de Zaragoza. Ctra. Huesca s/n, 50090 Zaragoza, Spain; 5 Department of Physics, Chemistry and Biology (IFM), Linköping University, SE-58183, Linköping, Sweden; 6 Materials Modeling and Development Laboratory, National University of Science and Technology “MISIS”, Moscow, 119049 Russia; 7 ALBA-CELLS, 08290 Cerdanyola del Vallés, Barcelona, Spain; 8 Ehime University, 2–5 Bunkyo-cho, Matsuyama, 790-8577 Japan; 9 Earth-Life Science Institute, Tokyo Institute of Technology, Tokyo, 152-8500 Japan

**Keywords:** Condensed-matter physics, Condensed-matter physics

## Abstract

The 5d transition metals have attracted specific interest for high-pressure studies due to their extraordinary stability and intriguing electronic properties. In particular, iridium metal has been proposed to exhibit a recently discovered pressure-induced electronic transition, the so-called core-level crossing transition at the lowest pressure among all the 5d transition metals. Here, we report an experimental structural characterization of iridium by x-ray probes sensitive to both long- and short-range order in matter. Synchrotron-based powder x-ray diffraction results highlight a large stability range (up to 1.4 Mbar) of the low-pressure phase. The compressibility behaviour was characterized by an accurate determination of the pressure-volume equation of state, with a bulk modulus of 339(3) GPa and its derivative of 5.3(1). X-ray absorption spectroscopy, which probes the local structure and the empty density of electronic states above the Fermi level, was also utilized. The remarkable agreement observed between experimental and calculated spectra validates the reliability of theoretical predictions of the pressure dependence of the electronic structure of iridium in the studied interval of compressions.

## Introduction

Iridium (Ir), with electronic structure [Kr]4d^10^5s^2^5p^6^4f^14^5d^7^6s^2^, is one of the most incompressible 5d transition metals with face-centered cubic (fcc) structure. It is the second densest elemental metal having an ambient pressure density of 22.65 g/cc at T = 0 K and 22.56 g/cc at T = 293.15 K, and a shear modulus, G_o_ = 210 GPa, comparable to that of osmium, G_o_ = 220 GPa, at ambient conditions^1^. On top of that, Ir has an extremely high thermal stability, being able to preserve mechanical stability at temperatures above 2000 °C and it is not easily susceptible to corrosion. These properties make Ir useful for many technological applications; for instance, as a high-pressure gasket or as a pressure calibrant in high temperature and high-pressure (HP) diamond-anvil cell (DAC) experiments. On the other hand, Ir is used for the construction of thermocouples and encapsulators of nuclear-powered electrical generators in space technology^2^. Furthermore, Ir is being currently used to synthesize Ir-based double perovskite compounds and iridium-based transition metal oxides or iridates, and its application fields stand from high-temperature superconductivity^3^, magnetoresistance^4^, to multiferroicity^5^. Whereby, its use in numerous applications make the study of Ir metal of great interest from a fundamental standpoint.

The study of the 5d metals under pressure have attracted the attention of the scientific community since the beginning of the 21^st^ century because of the relevance of their behaviour under extreme conditions for improving the knowledge of planet interiors^6^. In this sense, the debate on the structural stability of iridium under pressure has taken years. In 2000, Cerenius *et al*.^7^ reported the formation of a complex superlattice in iridium above 59 GPa in a high-pressure energy-dispersive x-ray diffraction (XRD) experiment. The structure of such a superlattice corresponded to a 14-layer hexagonal closed packed structure with lattice parameters a = 2.60 Å and c = 29.68 Å. In subsequent experimental-theoretical studies, such a phase transition was not predicted^8,9^. More recent studies have shown that 5d metals undergo pressure-induced peculiarities in their electronic structure. L. Dubrovinsky *et al*.^10^ have reported a new type of electronic transition, the so-called core-level crossing (CLC) transition in osmium (Os) metal at around 440 GPa. This pressure-induced transition has been associated with interactions between the core electrons that affect the pressure evolution of the lattice parameter ratio c/a in Os at 440 GPa. In a methodical theoretical of the CLC transition reported the prediction of a generality of the effect in 5d transition metals^11^, revealing a CLC transition for Ir at a reasonable low-pressure of 80 GPa. Such a prediction makes Ir a great candidate for the study of its pressure-induced evolution of the electronic structure, in which the 5p and 4 f core electrons at the CLC transition could affect the valence electrons due to the nonlocal nature of the electron interactions. Unfortunately, the effect of the CLC transition on the valence states is expected to be extremely weak and an observation of its influence on structural, thermal, electronic and transport properties of the metals might represent a true challenge^10^. Consequently, it is crucial to perform an exhaustive characterization of the stability and the electronic structure of compressed Ir metal at the megabar range.

In this work, we clarify the structural stability, accurately determine the P-V equation of state (EoS) in the pressure range up to 1.4 Mbar, and report the high-pressure phase of Ir through a detailed quasi-hydrostatic XRD and x-ray absorption (XAS) study in which the effect of the pressure on the electronic structure of the Ir is explored. In hexagonal-closed-packed (hcp) metals, such as Os^10^, the CLC can be detected by XRD through changes in the c/a ratio; this is not the case of Ir, with fcc structure. Furthermore, the crossing of the 4f and 5p orbitals in the core could affect the 5d electrons due to the nonlocal potential interaction, an interaction expected to be very weak. Thus, in order to explore the occurrence of a CLC in Ir we have carefully explored any change of compressibility or in the local structure. Despite of the weak effect searched, the occurrence of the CLC in Ir could lead to an energy shift, a broadening of the band, or a change of intensity of the white-line (WL) in the x-ray absorption near edge structure (XANES). Thus, besides obtaining careful information of the local structure of Ir, we attempt to use XAS to obtain information about the electronic and thermal effect of the CLC in this metal.

## Results and Discussion

### X-ray diffraction

**Figure 1:**
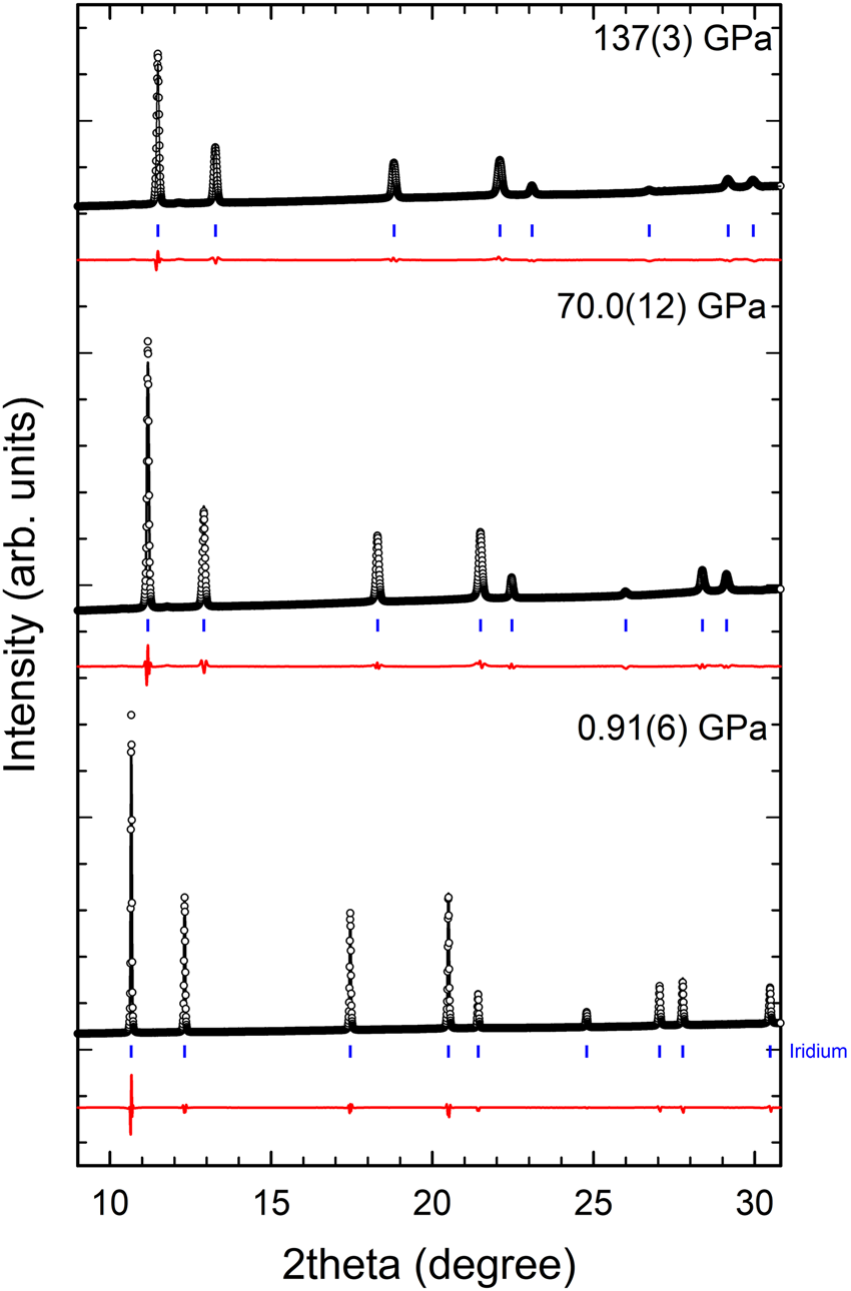
Rietveld refinements of diffraction patterns at 0, 70 and 137 GPa. Black empty circle line corresponds to the measured XRD pattern, black (red) solid line shows the refinement (residual) and the blue ticks are the position of Bragg peaks of Ir.

Ir metal crystallizes in the space group Fm $\bar{3}\,$m, an fcc structure, with lattice parameter at ambient pressure of 3.837(1) Å. The fcc structure was used in the Rietveld refinements up to 1.4 Mbar, showing no evidence of phase transition or structural distortion, in contrast with the results obtained by Cerenius and Dubrovinsky^7^. Fig. 1 shows the refinements to three different diffraction patterns at 0, 70 and 140 GPa, where the reflections of the superlattice exhibited by Cerenius and Dubrovinsky are not observed. Here, it is important to underline that our experiments were carried out in quasi-hydrostatic conditions, so that the appearance of the mentioned reflections in the XRD patterns measured by Cerenius and Dubrovinsky could be due to the non-hydrostaticity of the PTM (magnesium oxide, MgO) used^12^. Indeed, L. Burakovski *et al*.^9^ had already concluded theoretically that such phase transition was caused by the lack of hydrostaticity during the experiment. Therefore, it is clear that no phase transition and no structural distortion are observed in our diffraction data.

**Figure 2:**
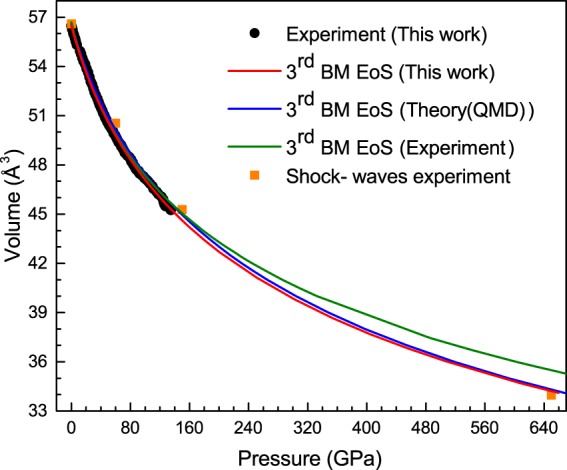
Experimental pressure-volume curve obtained by our XRD experiments (black points) and shock-waves data (ref.^14^) (orange squares). The 3^rd^ order EoS are represented by solid lines: red line for our data, blue line for the values calculated by quantum molecular dynamics (QMD) (ref.^9^) and green line for the data obtained by Cerenius and Dubrovinsky (ref.^7^).

The pressure-volume (P-V) curve (Fig. 2) shows a smooth volume decrease. Often, electronic transitions affect the structure of materials resulting into changes in the compressibility and local environment of the atoms^13^. However, we do not see any clear discontinuity in the P-V curve. We fit our experimental data to third order BM EoS, which is very similar to that obtained from previous *ab initio* calculations up to 900 GPa by L. Burakovski *et al*.^9^ (Table 1). The order of the EoS has been determined by the positive linear slope exhibited by the F-f (Eulerian strain-normalized pressure) plot of the theoretically simulated volume vs pressure curve, not shown here. The *ab initio* calculations and our quasi-hydrostatic experiments agree very well, which demonstrates that this is the most appropriate EoS order for the compressed Ir metal. Moreover, an extrapolation to 650 GPa totally agrees with shock-wave experiments (Fig. 2)^14^. Although, it is well known that dynamic high-pressure experiments involve a temperature increase under compression, in the case of Ir the effect of temperature can be neglected.

**Table 1:** Ambient volume V_0_, bulk modulus B_0_, first derivative of bulk modulus B_0_′, maximum pressure reached, P_max_, pressure transmitting medium (PTM) and calibrants.

V_0_ (A^3^)	B_0_ (GPa)	B_0_′	P_max_ (GPa)	PTM	Calibrant	
56.48	339 (3)	5.3 (1)	137	He	Cu	This work
56.58	383 (14)	3.1 (8)	60	Ar	Ruby	ref.^1^
56.58	366	5.0	*Ab initio* up to 900 GPa			ref.^9^
56.69 (17)	306 (23)	6.8	65	MgO	MgO	ref.^7^

Note that the third-order BM EoS from Cerenius and Dubrovinsky underestimates the bulk modulus, B_0_ = 306(23) GPa, and overestimates its derivative, B_0_′ = 6.8(1.5) (Table 1). In fact, an extrapolation to very high pressure, above 600 GPa, does not reproduce the experimental results from shock-waves experiment (Fig. 2).

### X-ray absorption spectroscopy and *ab initio* calculations

**Figure 3:**
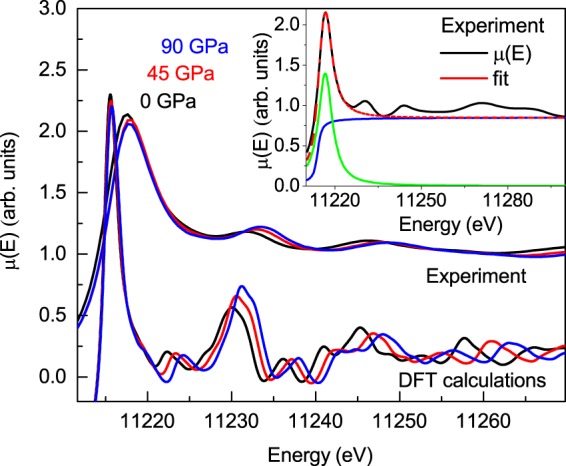
Experimental (simulated) x-ray absorption spectroscopy (XAS) spectra at ambient pressure, at 45 GPa and 90 GPa in black line, red line and blue line, respectively. The Lorenztian + arctang fit of the WL (experimental spectrum at room pressure) is represented in the inset. Red dash line is the total fit of the WL, the blue line corresponds to the arctangent fit function and the clear green line is the lorenztian fit function.

**Figure 4:**
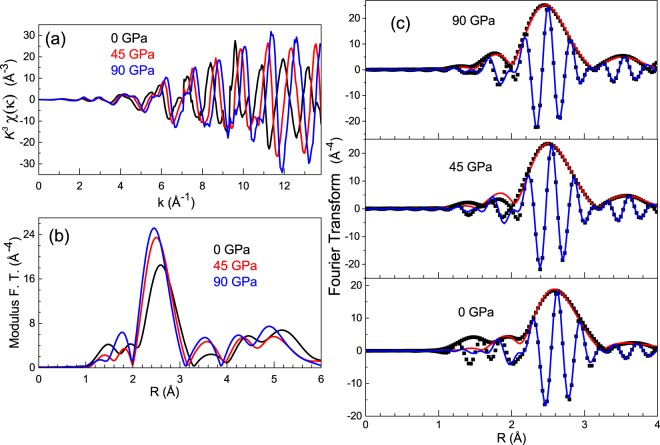
(**a**) k^3^χ(k) EXAFS signals and (**b**) Fourier Transform at the Ir L_3_-edge at 0 GPa (black curve), at 45 GPa (red curve) and at 90 GPa (blue curve). (**c**) The Fourier transformed k^3^χ(k) EXAFS signals at 0 GPa, at 45 GPa and at 90 GPa are represented in black squares. The finest fits of the modulus and imaginary parts are shown in red and blue line, respectively.

X-ray absorption (XAS) measurements were collected at the Ir L_3_ absorption edge (11.215 KeV) and are shown for three selected pressures in Fig. 3. These spectra can be decomposed into three main contributions: (i) a sharp, atomic-like “white-line” that can be assigned to the 2p_3/2_ → 5d electronic transition, (ii) a step-like edge associated with 2p_3/2  _→ continuum electronic excitations, and (iii) smaller oscillations (fine structure) that result from the photoelectron backscattering from neighboring atoms. The EXAFS signal weighted in *k*^3^ and the modulus of its Fourier transform (FT) at pressures of 0, 45 and 90 GPa are presented in Fig. 4a. The EXAFS signals exhibit a worthy quality up to *k* = 13.4 Å^−1^. The FT of the *k*^3^*χ*(*k*) weighted EXAFS signals were calculated in the interval *k = *4–13.4 Å^−1^ using a Hanning window, as represented in Fig. 4b,c. The FEFF-8 code^15^ was employed for the structural analysis. The modulus and imaginary part of the FT (black symbols) are shown in Fig. 4c at 0, 45 and 90 GPa. The best-fittings (red and blue solid lines) are also represented in such a figure. First and second neighbouring shells have been included in the analysis, which was performed using the Artemis code in R-space, between 1.8 and 4 Å^−1^. The nearest- and second-neighbour distances were modelled following one single-peak average distribution, fixing the coordination numbers to the theoretical values. As a consequence, there were only four fitting parameters: the Debye-Waller (DW) factor (σ^2^) for both average distance distributions, the deviance from the average distance (ΔR), the amplitude reduction factor (S_0_^2^), and the edge-energy difference between experiment and theory (ΔE_0_). S_0_^2^ was determined at ambient conditions and the obtained value of 1.0(2) was considered to be pressure independent. A similar procedure was used for ΔE_0_ getting a value of 8(2) eV.

**Figure 5:**
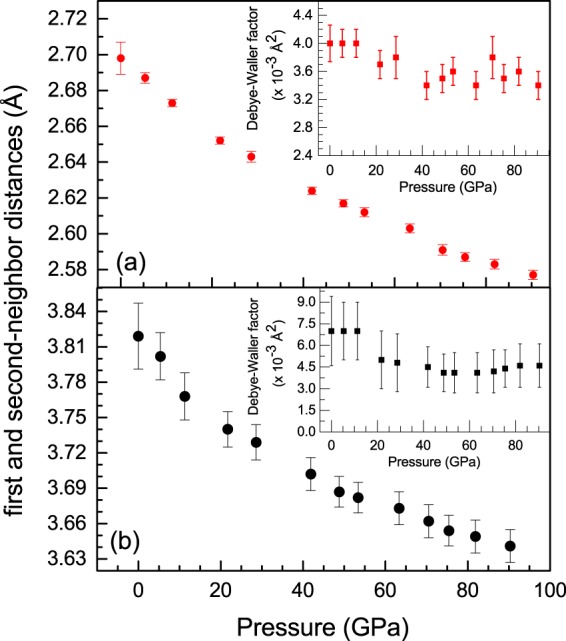
(**a**) Pressure evolution of the nearest-neighbor distances and (**b**) second-neighbor distances. The Debye-Waller (DW) factors that correspond to the first and second shell are represented in the insets.

The first more intense peak in the EXAFS Fourier transform is associated with the first shell around Ir atoms while the second corresponds to the second shell. The starting modelization assumed for the fitting of the FT signals, in the full pressure range studied, includes the nearest-neighbor Ir-Ir distances of the dodecahedra and the second-neighbor Ir-Ir distances of a hexagonal environment, both associated to an fcc structure with a stacking sequence of layers ABC. The first and the second-neighbor distances have a smooth evolution under pressure without remarkable changes that could be associated to any electronic transition (Fig. 5(a,b)).

No significant changes are observed, within the error bars, in the pressure evolution of the DW factors corresponding to the first and the second shell (insets of the Fig. 5(a,b)). The DW factors corresponding to the first-neighbor distances maintain a constant value around 0.0035 Å^2^. The DW factor for the second shell is around 0.007 at ambient pressure and it remains around this value as pressure increases. The DW factor includes a thermal contribution due to the local vibrational dynamics and a structural contribution linked to the local structural distortions. In relation to the structural contribution, the first shell around the Ir atoms is almost regular and the higher value of the DW factor of the second shell could reflect a more distorted environment. Now, considering only the thermal part of the DW factor, we can try to understand whether this is a signature of the occurrence of the CLC since the electronic transitions, while not affecting explicitly lattice parameters, are expected to cause anomalies in the behavior of the vibrational and transport properties.

Within the framework of the harmonic approximation for a cluster with N atoms, using Bose-Einstein statistics and considering only single scattering paths, the DW factor depends on the phonon frequencies as follows^16^:

1 $${\sigma }_{j}^{2}(T)=\,\frac{{\rm{\hbar }}}{2{\mu }_{j}}\,{\int }_{0}^{{\omega }_{max}}\frac{d\omega }{\omega }\,{\rho }_{j}(\omega )coth(\frac{\beta {\rm{\hbar }}\omega }{2})\,$$

where μ_j_ is an effective reduced mass for scattering path *j* that guarantees normalized initial conditions. The parameter $\beta =1/{k}_{B}T$, ${\rho }_{j}(\omega )$ is a projected density of vibrational modes. It weights the contribution of every mode to the specific vibrational motion along the bond direction. In addition, ${\omega }_{max}\gtrsim z\,\sqrt{{k}_{1}/{\mu }_{1}}$ is the maximum frequency of the lattice motion. It depends on the coordination number, *z*, the central first-neighbor force constant, ${k}_{1}$, and ${\mu }_{1}$ is the half of the mass of the iridium atom^16^. The short wavelength modes are those that dominate the vibration of the first and second neighbor atoms since vibrational energies involved are typically between 300 and 3000 cm^−1^. However, from the similarity of the effect of CLC and more well studied electronic topological transition (ETT) on the pressure dependence of c/a axial ratio in hcp Os^10^, it is expected that the former would imply an anomalous behavior in the long wavelength phonon modes, which has been discussed in detail by Glazyrin *et al*.^17^.

Therefore, we conclude that the DW factors are unlikely to bear a signature of the effect of the CLC transition, which is also evident if we pay attention to the stability of these values in the insets of the Fig. 5(a,b).

The most striking feature of Ir L_3_ edge XANES is the prominent white-line (WL) observed. The integrated intensity (or area) of the white-line peak is correlated with the local density of unoccupied final states in the system^18^. The intrinsic width of the 5d band of the compound is the parameter that mainly determines the linewidth. However, it is also influenced by broadening mechanisms which include core-hole lifetime effects, final-state lifetime effects, and obviously instrumental resolution^18,19^. These observations are consistent with conclusions from a systematic work of Ir L_3_ lineshapes in a series of Ir-based oxides^20^. Another consideration to take into account is the strong spin-orbit coupling (SOC) effect in the Ir metal and also in different iridates such as Sr_2_IrO_4_, Na_2_IrO_3_ and Y_2_Ir_2_O_7_. J. P. Clancy *et al*.^21^ have reported the spin-orbit expectation value $\langle {\rm{L}}\,\cdot \,{\rm{S}}\rangle$ of these materials, which are −1, −3.1, −2.7 and −2.8 expressed in units of ${{\rm{\hbar }}}^{2}$, respectively. Therefore, the SOC energies are comparable to the crystal field and the Coulomb electronic interaction in these systems, and therefore it plays an important role on the electronic ground state, as will be discussed below.

**Figure 6:**
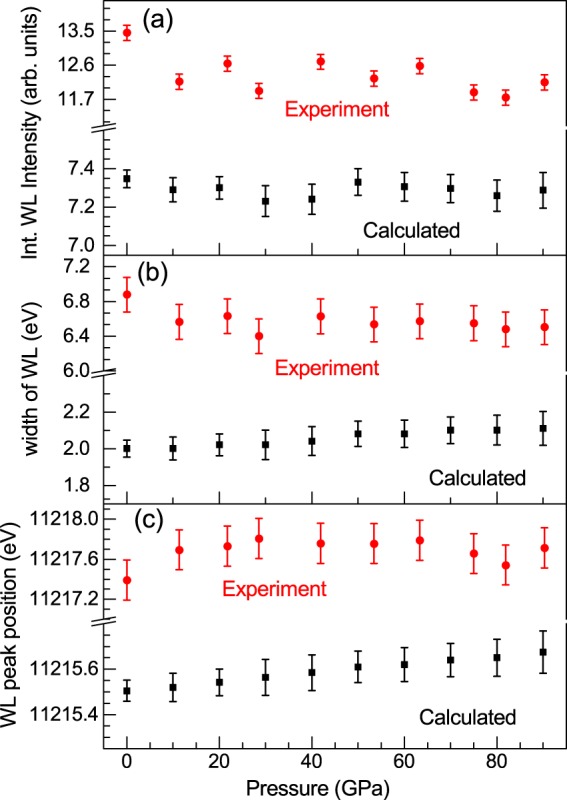
(**a**) Integrated intensity of WL of the XAS spectra collected at Ir L_3_ edge under pressure is represented, (**b**) the pressure evolution of the linewidth of the WL is plotted and (**c**) the variation of WL peak position under pressure is shown. Red points correspond to experimental data and black points correspond to simulated data using WIEN2k code.

The integrated intensity of the white-line at the L_3_ edge and its linewidth are obtained through an arctangent + Lorentzian fit function as can be seen in the ambient condition experimental XAS spectrum in the inset of the Fig. 3. The pressure evolution of these values, in all the pressure range studied is shown in Fig. 6(a,b). There are no dramatic changes neither on the integrated intensity of the WL nor on its linewidth along the whole pressure range. The intensity of WL decreases slightly as the pressure increases and the linewidth keeps almost constant. These experimental results have been compared with those calculated from first principles. The simulated XANES spectra can be seen in Fig. 3 and it reproduces very well the experimental ones. Both experimental and theoretical integrated intensity and linewidth have the same trend under pressure as shown in Fig. 6(a,b). Moreover, the experimental and theoretical WL peak position has been represented in Fig. 6c and no anomalies are observed.

Neither theoretical nor experimental results bear observable signatures of the CLC. In order to give an explanation of why the CLC effect is not appreciable in the XANES of Ir L_3_ edge, let us consider all the energies involved in the process. The full Hamiltonian of a system is:

2 $$\hat{H}={\hat{H}}_{0}+{\hat{H}}_{SO}+{\hat{H}}_{CF}$$

The first term ${\hat{{\rm{H}}}}_{0}$ takes into acount the kinetic energy, $\hat{{\rm{T}}}$, and the interaction potential, $\hat{{\rm{V}}}$. Within the Phillips-Kleinman pseudopotential approximation, the interaction potential can be divided in two contributions $\hat{{\rm{V}}}=V+{\hat{{\rm{V}}}}^{R}$ being *V* an effective potential that operates on the electron and ${\hat{{\rm{V}}}}^{R}$ the non-local operator that acts on the smooth part of valence orbitals in this way:

3 $${\hat{{\rm{V}}}}^{R}\,{\tilde{\psi }}_{i}^{v}(\overrightarrow{r})=\,\sum _{j}({\varepsilon }_{i}^{v}-{\varepsilon }_{i}^{c})({\psi }_{j}^{c}|{\tilde{\psi }}_{i}^{v}){\psi }_{j}^{c}(\overrightarrow{r})$$

However, this potential, $\hat{{\rm{V}}}$, is weak.

The second term corresponds to the spin-orbit interaction obtained from the Dirac equation and it is written as

4 $${\hat{{\rm{H}}}}_{{\rm{SO}}}=\xi (r)({\rm{L}}\cdot {\rm{S}})$$

where $\xi (r)=\,-\,\frac{e\,{{\rm{\hbar }}}^{2}}{2{m}^{2}{c}^{2}}\frac{1}{r}\,\,\frac{dU(r)}{dr}$, with a potential $U(r)$ for the electron that is spherically symmetric. As mentioned above, the SOC in Ir metal is strong and leads to important effects at the Ir L_3_ edge WL.

The last term, the crystal field (CF) potential, describes the interaction of the electrons of central atom with these of ligands. It strongly depends on the symmetry of the crystal structure. In general, such a contribution to the electron potential energy is given by

5 $${V}_{CF}(\overrightarrow{r})=\sum _{i=1}^{N}\frac{Z\,{e}^{2}}{\overrightarrow{{R}_{i}}-\overrightarrow{r}}$$

where $\overrightarrow{{R}_{i}}$ is the position vector of the *i*-th point charge, $\overrightarrow{r}\,\,$is the electron coordinate and the summation includes all the point charges. In the 5d transition metals, the crystal field values are high, between 1 and 5 eV, hence the CF effect is also significant at the Ir L_3_ edge WL.

Therefore, and according to the theory, the effect of CLC cannot be strong in the XANES of the Ir since the non-local potential, which acts on the valence electrons, and is modified due to substantial reconstruction of inner 5p and 4f states at the transition, is very weak compared with the strong SOC and CF effect in Ir. On the other hand, the excellent agreement achieved between experimental and calculated spectra supports the accuracy of theoretical predictions of the behavior of the electronic structure of Ir under compression in the studied interval of compressions.

## Methods

### X-ray diffraction

The HP-XRD experiments were carried out at ID15B beamline of the European Synchrotron Radiation Facility (ESRF, Grenoble, France). A monochromatic x-ray beam (E = 30 keV, λ = 0.4111 Å) was focused on the sample using two sets (one horizontal and one vertical) of compound refractive lenses^22^ to a beam size of 7 × 7 µm^2^. The XRD patterns have been collected using a MAR555 amorphous Se flat panel detector located at 400 mm from the position of the sample. Ir powder and a small amount of copper (Cu) powder, to be used as a standard pressure^23^, were placed in the pressure chamber of a membrane-driven DAC, mounting Boehler-Almax-design diamonds (culet diameter: 150 μm). The pressure chamber (50 μm in diameter) was laser drilled in a Re gasket, which were previously indented to 15 μm. The PTM selected was He to ensure the best possible hydrostatic conditions during the measurements. At each pressure, two images were collected, one in the position of the sample and another one in the position of the Cu powder. In each acquisition the ω-axis of the DAC was rotated between −3° and +3° in a single step with 1 second exposure time. The patters were integrated using DIOPTAS software^24^ and the pressure was calculated using the equation of state proposed by Dewaele *et al*. for the Cu powder. Data analysis was conducted by GSASII software^25^. The integrated x-ray diffraction patterns were fitted by Rietveld refinement method, but the lack of free coordinates of the atomic position allowed to determine the lattice parameters. These structural data have been fitted to a Birch-Murnaghan equation of state^26^ (BM EoS) using the software EosFit7^27^.

### X-ray absorption

The experiment has been performed at the BM23 beamline^28^ of the ESRF armed with a double crystal Si(111) monochromator and Kirkpatrick−Baez mirrors with a Pt coating to concentrate the x-ray beam to 4 × 4 μm^2^. The mirrors were fixed to an angle of 5 mrad to eliminate high order harmonics. XAS was measured in transmission mode at Ir L3-edge (11.215 KeV) until 90 GPa using a membrane-type pressure cell (DAC) equipped with 150 μm culet nanopolycrystaline diamond anvils^29^. The pressure transmitting medium (PTM) was helium (He) and we determined the pressure through the luminescence of a ruby placed in the cell.

The Extended X-ray Absorption Fine Structure (EXAFS) signal was extracted in a conventional way using the Demeter package^30^. In addition, the X-ray Absorption Near Edge Structure (XANES) was studied by carrying out simulations of XANES spectra in the framework of Density Functional Theory (DFT) using the WIEN2k software package^31^. Here, the Generalized Gradient Approximation exchange-correlation function PBE according to Perdew *et al*.^32^ was utilized. WIEN2k is based on the highly accurate all-electron Full-Potential Linearized Augmented Plane-Wave (FPLAPW) method. In this case, the Augmented Plane-Wave plus local orbital (APW + lo) basis set was used and spin-orbit coupling was included. To account for final state effects in the Ir L_3_ XANES spectra, a core-hole was inserted at the 2p_3/2_ orbital for a single atom. To avoid artificial interactions between core-ionized sites due to periodic boundary conditions, supercells of 108 Ir atoms were chosen for the calculations. By promoting a single electron to the valence band, the charge neutrality of the supercells was preserved. Lattice parameters were selected as corresponding to experimental values for the different pressures. While a single k-point was sufficient for the self-consistent DFT supercell calculations, an extra step with an 8 × 8 × 8 k-point mesh was done before obtaining the XANES spectra using the XSPEC package within WIEN2k. Finally, a 1.5 eV full width at half maximum Lorentzian broadening was applied for comparison with experiment.

## Conclusions

Ir metal has been studied by two complementary techniques, XRD and XAS, as well as by theoretical DFT calculations in order to characterize its high-pressure structure and electronic properties. Ir metal remains in the fcc structure in the full pressure range studied. We characterized its pressure-volume EoS up to 1.4 Mbar using a third order BM EoS, giving rise to an experimental bulk modulus at ambient pressure of 339(3) GPa and its derivative of 5.3(1). Experimental characterization of its structure confirms the large stability of the low-pressure phase. The exhaustive analysis of the local structure and the electronic structure associated to the 2p → 5d transition, revealed the decrease of the first and second-neighbor distances while DW factors remain almost constant under compression. Moreover, the pressure dependence of the integrated intensities, the width and the peak position of the WL does not present remarkable changes. Here, we have demonstrated the difficulty to detect the effect of the CLC transition in the Ir metal through both experimental techniques. Nevertheless, we observe a nice agreement between experimental and theoretically calculated structural and electronic results. This feature supports the reliability of the theoretically predicted CLC transition in Ir at 80 GPa, which implies that additional experimental paths are needed to unambiguously verify its presence.
